# Supplementary material for: Clinical Impact of Supraclavicular Lymph Node Involvement of Stage IIIC Non-Small Cell Lung Cancer Patients
Source: Medicina (Kaunas). 2021 Mar 23;57(3):301. doi: 10.3390/medicina57030301 (PMC8004859; doi:10.3390/medicina57030301)
Supplement: Supplementary file 1 [file medicina-57-00301-s001.pdf]

Supplementary Table S1. Clinical characteristics between patients with and without supraclavicular lymph node (SCN) involvement

	SCN (-) (N=23)	SCN (+) (N=17)	P value
Performance status			0.546
0 or 1	19 (82.6%)	16 (94.1%)	
2 or higher	4 (17.4%)	1 (5.9%)	
Age			
< 60	5 (21.7%)	4 (23.5%)	1.000
≥ 60	18 (78.3%)	13 (76.5%)	
Sex			
Male	21 (91.3%)	11 (64.7%)	0.093
Female	2 (8.7%)	6 (35.3%)	
Smoking			
Non-smoker	2 (8.7%)	5 (29.4%)	0.199
Smoker	21 (91.3%)	12 (70.6%)	
Histology			
Other histology	6 (26.1%)	8 (47.1%)	0.299
Squamous cell carcinoma	17 (73.9%)	9 (52.9%)	
T stage			
1 or 2	12 (52.2%)	5 (29.4%)	0.264
3 or 4	11 (47.8%)	12 (70.6%)	
RT modality			
3DCRT	15 (65.2%)	6 (35.3%)	0.120
IMRT	8 (34.8%)	11 (64.7%)	

Abbreviations: SCN, supraclavicular lymph node; RT, radiotherapy; 3DCRT, 3-dimensional conformal radiotherapy; IMRT, intensity-modulated radiotherapy

Supplementary Table S2 Toxicity parameters and radiation profile according to the radiation therapy modality

	3DCRT (N=21)	IMRT (N=19)	P value
Grade ≥3 pneumonitis			0.042
No	20 (95.2%)	14 (73.7%)	
Yes	1 (4.8%)	5 (26.3%)	
Grade ≥3 esophagitis			0.928
No	20 (95.2%)	17 (89.5%)	
Yes	1 (4.8%)	2 (10.5%)	
Grade ≥2 esophagitis			1.000
No	9 (42.9%)	8 (42.1%)	
Yes	12 (57.1%)	11 (57.9%)	
Grade ≥2 pneumonitis			1.000
No	15 (71.4%)	13 (68.4%)	
Yes	6 (28.6%)	6 (31.6%)	
EQD2 (continuous)	60.6 ± 6.0	64.2 ± 7.9	0.105
EQD2			0.004
<63 Gy	15 (71.4%)	5 (26.3%)	
≥63 Gy	6 (28.6%)	14 (73.7%)	
Volume of CTV	294.0 ± 152.7	439.0 ± 287.1	0.060
Mean lung dose	1238.8 ± 388.6	1655.8 ± 579.1	0.010
V20	23.9 ± 8.3	31.6 ± 19.0	0.114

Abbreviations: 3DCRT, 3-dimensional conformal radiotherapy; IMRT, intensity-modulated radiotherapy; CTV, clinical target volume; V20, volume of the normal lung receiving a dose of 20 Gy.
